# Dr. Paxton's Case of Abscess in the Liver

**Published:** 1812-02

**Authors:** 


*To the Editors of the Medical and Physical Journal.*

GENTLEMEN,

AMONGST the papers of Dr. Paxton, a physician some years since deceased at Maldon, in Essex, I found the following history of a case, which appears to me worthy of insertion in your Journal.

I am, Gentlemen,
Your obedient Servant,
An ESSEX PRACTITIONER.

"The writer was first called to Mr. R. on the 8th of November, 1787. He was then very ill, labouring under strong symptoms symptoms of an autumnal intermittent, which, however, by the use of emetics, laxatives, and the usual mild antimonials, seemed nearly removed in three or four days. On the 13th, finding himself worse, he sent for a physician, who saw him next day. He directed saline medicines, joined with mild evacuants, and pronounced him free from danger. Being a farmer, he was soon about to ride in his fields: some small degree of fever, however, still subsisted, and he complained of an obtuse or dull pain on the right side, near the ends of the false ribs. In this state he continued to the end of December, on the 29th of which we* were again called to him. In his own idea he had had that day a severe fit of ague, and expected a return on Monday, that being Saturday evening. We agreed upon an emetic to be taken at the access of the expected fit, and after it a purgative. The fever not subsiding, but continuing to increase, before he took the emetic, he sent for Dr. B. who approved of what had been prescribed, but added to it the effervescing powders, one to be taken every four hours, in an infusion of colomba, with lemon juice. On the 2d of January, 1788, he directed a repetition of the emetic and purgative; the powders and mixture to be continued. On the 4th he ordered the following ℞. Sal. Absinth. ʒiiſs. Suc. Lim. ℥v. Decoct. Peruv. ℥viiſs. Pulv. Colomb. gr. xxv. Tart. Solub. ʒiiſs. Tinct. Peruv. ℥ij. M. Sumat. Coch. iij. ter in die. This was repeated on the 6th, omitting the Tart. Solub. and occasionally substituting a few drops of Tinct. Opii. On the 8th he was somewhat better; the purgative pills were repeated, and his medicine was changed for a spermaceti emulsion, with Pulv. Myrrh, Vin. Ant. and Tinct. Opii, and a bitter infusion as a mild corroborant was added to his regimen. 14th. He was worse, and a tendency to diarrhœa supervening, he had the following ℞. Pulv. Ipec. ℈j. Tinct. Japon. ʒj. Opii. gr. ij. Aquæ ʒvj. Mft. haust; this not operating, we gave him ℥j. of Vin. Ipecacuan. and afterwards a bark mixture with Tinct. Opii, a dose to be taken three times a-day. 16th. ℞. Sal. Tart. gr. x. Cret. Præp. gr. iv. Mft. pulv. in actu effervescentis cum coch. iij. Misture seq 6ta quaque horâ sumendus. ℞. Decoct. Peruv. ℥viij. Suc. Lim. ℥vj. Tinct. Peruv. ℥ij. Opii gtt. xv. Syrup Mecon. ʒvj. M. Still the slow fever, cough, and a constant degree of pain in the hepatic region, existed. 19th. He took a dose of pills made with Pulv. Rhæi. gr. xij. Calomel gr. iv. and continued the mixture and powders. 21st. The pills

* The writer was in partnership with another surgeon.

were were repeated, and to each powder was added 5 grains of Pulv. Calomb. 23d. ℞. Calomel gr. j. Opii gr. ſs. ft. Pilula bis in septimana sumenda. ℞. Gum Myrrh ʒj. Sal Tart. ʒſs. Sal Martis gr. xv. Aq. Cort. Aur. ℥iſs. Aq. Puræ ℥vj. Syrup Mecon ʒvj. M. Capt. Coch. iij. bis in die. 24th. A seton was made on the hepatic region, where the pain was. In the evening of this day, a new and unexpected scene presented itself. He had found himself during the day no worse than usual. He sat up three hours in the afternoon, and went to bed about seven in the evening; and soon after, according to his own expression, he heard something snap within him; at the same instant a violent cough came on, with a copious expectoration of matter, purulent and very offensive. He immediately sent for us, and we found him bringing up by the mouth large quantities of pus. It was deemed right that Dr. B. should be acquainted with what had happened, and be requested to attend immediately. He came about one in the morning, by which time our patient had discharged into a bason about five pounds of pus. The quantity now in a given time lessened, and was gradually brought up by the cough. The doctor pronounced that the discharge proceeded from an abscess of the liver, to which we could not then assent. The doctor was further of opinion that he could not survive 48 hours. He recommended a continuance of the myrrh mixture, and further prescribed Decoct. Cinchon. cum Conf. Card. The patient was so far from being in a dying state, that the next day he appeared revived, continued his medicines, and expectorated moderately. On the following day, a little purging supervening, 15 grains of Creta were added to every dose of his medicine. Dr. B. called upon us this day, and was not a little surprised to hear that Mr. R. was not only alive, but better. He still persisted that the discharge proceeded from an abscess of the liver, though we confess we were not satisfied with his reasons; but his opinion was perfectly right, as appeared afterwards. The four succeeding days Mr. R. continued nearly in the same state, expectorating about a pint of pus every 24 hours. At two o'clock in the morning of the 30th he was attacked with a second irruption of pus, and in five hours he discharged 3 pounds. Intelligence of this was communicated to Dr. B. who, by a letter, expressed his opinion that another cyst had been ruptured; and recommended a continuance of the peruvian and chalybeate medicines, with a plentiful supply of light nourishing diet. Indeed he was not only allowed, but anxiously desired, to drink often a glass of some generous white wine, plenty of porter to his meals, and any thing of the kind that would agree, in

 order, order, if possible, to support the strength under such a vast discharge. By the early part of next day he had raised up 3 pounds more. The doctor saw him, ordered a continuance of his medicines, with the following ℞. Spermaceti ℥ſs. Vitel. Ovi. q.s. Lac. Ammon. ℥iij. Elix. Pareg. ℥j. Aq. Cort. Aur. ℥ij. Aq. Puræ ℥viij. Syr. Mecon ℥j. M. Sumat Coch. iij. frequenter.

Feb. 1st to 10th. He continued much in the same state, expectorating daily a pound or more of pus, very offensive; pulse from 90 to 100, which was its general standard throughout the disease. He had no rigors, and seldom any exacerbations, slight sweats, and generally a soft moist skin. He took plenty of nourishment, yet by this time he was reduced almost to a skeleton, and had the same appearance as a person in the last stage of a consumption. The chalybeate medicine was continued, and occasionally the emulsion, omitting the lac ammoniac. The bark mixture was also omitted. Indeed the bark, though given in various forms from first to last, hardly ever agreed with him. From the 10th to the 14th the quantity of discharge was increased to 2 pounds daily. 17th. It was again reduced to one pound, but, from the afternoon of the 18th to the next morning, the discharge amounted to three pounds; and for the last six hours was greatly charged with pure bile. This was a demonstration of the seat of the disease, and fountain of the discharge; and I wrote to the doctor acknowledging the founded proofs of his opinion, and that we no longer doubted of an abscess of the liver. From the 16th he had taken the following pills: ℞. Pil. Sapon ʒiſs. Gum Ammon. ʒſs. ft. pilul. xl. Sumat j. Omn. nocte et rept. instante nocte urg. tussi. He passed a good night on the 20th, and to the end of February there appeared no material alteration in the symptoms; the expectoration or discharge being about a pound in 24 hours, and making the same bilious appearance.

March 1st, and few following days, he tried, together with his chalybeate mixture, small doses of fine bark in camomile infusion. 5th. ℞. Extr. Cicut. gr. iv. Opii gr. j. M ft. pilul. omni nocte sum. sumat etiam Pulv. Peruv. ℈j. ex coch. iij. mist. seq. ℞. Decoct. peruv. ℥viij. Tinct. peruv. ℥iij. Tinct. opii gtt. xxv. ft. Mistura. These medicines not agreeing, he had no more till the 9th, when he again tried the bark, with one grain of rhubarb in every dose; the pill to be continued every night. 21st. A chalybeate mixture was again ordered, with lac ammoniac. and tinct. opii. Mr. R. was now so much better, that our visits were less frequent. 27th. In the afternoon we were sent for in great haste. Our patient had walked out into his yard, and, after returning ing into the house, was taken with a sudden discharge of florid blood by the mouth. When we arrived, the discharge had abated, but his expectoration was colored with blood. We wrote an immediate account to Dr. B. of what had happened, and he returned the following prescription. ℞. Sacchar. saturn. gr. ij. Opii gr. fs. ft. bolus 6tis horis sumend. cum haust. seq. ℞. Decoct. Peruv. ℥ifs. Alum gr. viij. Elix. Vitriol gtt. xv. Syrup Mecon ʒfs. ft. haustus. Next day at ten he discharged ℥viij. of blood, and at six in the evening ℥xx more. Dr. B. saw him, ordered the bolus to be continued every six hours, with the following draught. ℞. Decoct. Cinchon. ℥ij. Alum gr. xv. Elix. Vitr. gtt. xxx. pro haust. 29th. Medicines continued. 30th. In the evening he discharged ℔ij. of blood; the boluses were continued with three grains of Sacch. Saturn. in each, and three large spoonfuls of the following mixture, instead of the draught. ℞. Decoct. Cinchon. ℥xv. Syrup Mecon. ℥j. Acid Vitr. pur. gtt. lx. Alum ʒj. Tinct. Opii gtt. xviij. M. 31st. He again discharged ℔ij. of blood. His medicines were continued. These repeated discharges of blood were every time sudden, and gradually ceased, only that the pus expectorated, which continued in the usual quantity (℔j. in 24 hours), was streaked with blood.

April 1st. Medicines were continued as before, and to obviate costiveness an opening mixture was ordered. 5th. Costiveness continuing, he was ordered the following pill. ℞. Aloes Soc. gr. iij. Gutt Gamb. Sap. Hisp. aa. gr. j. ft. Pil. sumend et Repet. p. r. n. The bark mixture was continued with gtt. lxx. of acid vitr. The next day we met his physician. He had now the true saturnine colic, with a total suppression of stools. ℞. Ol. Ricin. ℥iifs. Vitel. ovi q. s. Tinct. Sen. ℥ij. Aq. Puræ ℥viiifs. Sumat Coch. iij. omni horâ donec dejecerit alvus. 7th. In the morning I was called to him, with the information that he was in great pain, and without stools, though he had taken nearly two of the above oily mixtures. I carried a three-ounce viol of ol. ricini, of which he immediately took ℥j. and he was directed to take the remainder in two equal portions, at the distance of an hour, unless there were stools and an abatement of pain. Next morning Dr. B. saw him early. He then had less pain, though no copious alvine dejections were yet obtained. He had taken and retained the whole of the oil. He now took ℥ifs more, and the same quantity was repeated in an hour. The next day he took the following pills. ℞. Pil ex Colocynth gr. xxv. Calomel. gr. v. ft. pilul. at the same time persisting in the use of the ol. ricini. By these means were procured most copious and sufficient alvine evacuations; after which he took occasionally, as pain required, two of

 the the following pills. ℞. Extr. Cicut. gr. xl. Pil. Sapon. ʒj. ft. Pilul. xl. 4th. Mr. R. now resumed the use of the saturnine pills, which had been interrupted by the constipated state of the bowels. Three doses were taken in 24 hours, at intervals the bark mixture, and occasionally the ol. ricini. On the night of the 11th, and morning of the 12th, our patient had a return of the hæmorrhage, greater than ever, and on the last of those days seemed nearly exhausted; and scarcely a possibility remained of his surviving two days more. He continued the use of the saturnine pills, and the mixture, to the 14th. The two following days being in great pain, and without stools, he took only the pills, composed of opium and cicuta, and repeated doses of ol. ricini. 17th. In addition to the ol. ricini, he took ʒj. of Pil. Colocynth. in eight pills. 18th. The doctor ordered Ruspini's styptic to be procured; and, in the mean time, a bolus with ℈j. of alum in each, was given as often as he could bear it; and for his pain, occasionally, the saponaceous pill. 19th. Four draughts were sent to him, with ʒj. of the styptic in each. During the remainder of April he took only the oil and pills, to obviate pain and costiveness. By this time a paralytic affection of every limb had taken place, insomuch that he could scarcely move a leg. His hands and fingers were totally useless, continuing in any position they were placed in: without doubt, this was the effect of the sacchar. saturni. From the 27th of March to the 4th of April a (space of seven days), he took, in doses of two or three grains, 128 grs. From 11th to the 15th (4 days), 72 grs. In all 200 grs.

In thus detailing the state of our patient at this period, and the quantity of the sacch. saturn. taken by him in a given number of days, the writer has not the most distant intention to throw the least blame on Dr. B. On the contrary, he thinks, and with great probability believes, that, to this medicine thus given, the safety, and, in a great measure, the future recovery, of Mr. R. may be ascribed.

During the month of May, he was frequently obliged to take strong purgatives, and occasionally opiates. Expectoration was purulent, and in quantity about ℔j. in 24 hours, with very little appearance of blood or bile; and on the whole, with regard to the abscess, he was certainly better.

June 6th. A plaster composed of Emp. Cymin. et Opio was applied Scrob. Cord. and an opening electuary, with the following mixture, were prescribed. ℞. Aq. Menth. Simp. ℥ivss. Tinct. Sennæ. ℥iij. Tinct. Opii. gtt. cc. Syr. Mecon. ℥j. M. This mixture was repeated occasionally during the remaining part of his illness, finding that it obviated both pain and costiveness, the yet remaining consequences quences of the sacch. saturn. By the end of June the cure of the abscess was considerably advanced; he felt no pain in the hepatic region; the quantity expectorated was reduced to three or four ounces in 24 hours, yet the paralysis still remained in the same state.

On the 6th of July he had a small return of hæmorrhage, which soon ceased, yet the expectoration after it was much streaked with blood. He was alarmed, and sent for me; and was also desirous of trying the draughts with Ruspini's styptic, as before mentioned. He did so, repeating one every hour till he had taken twenty, and then, from an opinion that they were of no use, would take no more. We attended our patient once or twice a-week to the middle of the month of August; and, by that time, the abscess was nearly, if not quite, healed; the cough and expectoration ceasing, his appetite was good, he became more corpulent, and was sanguine in his expectations of regaining the use of his hands and legs, so as to be able to feed himself. His hopes were fully realized, by his recovering his usual health, after a considerable period of time.

It is now almost five years since the beginning of his illness: his health still continues perfect, enjoying nearly the free use of his extremities, and undergoing the fatigues of hunting or business with as much ease and pleasure as ever in the former part of his life.

*Maldon, Sept.* 12, 1792. RICHARD PAXTON.

In the preceding account we have omitted to observe, that, in the month of Sept. 1788, Mr. R. came and resided near us for some weeks, when, for the cure of his paralytic affection, electricity was repeatedly and vigorously used; and, as we have in a variety of cases seen the beneficial effects of it in paralysis, so we have no doubt that it was very instrumental in perfecting a cure in this case.

Ten years have elapsed since his cure was complete, in which time he has enjoyed uninterrupted good health."

"*Nov.* 9, 1798."

Here finishes Dr. Paxton's history of the case, to which he subjoined some observations, but of too unimportant a nature to occupy the pages of your Journal. Since copying the above, I have learned that the subject of the case is now living in the parish of Alton, near Maldon, enjoying good health and active exercise, being fond of field sports. His name is Mr. Rush. This case was probably published at the time in some periodical Journal; if so, I have not seen it, nor is it likely that many of your readers have.

